# Antimicrobial and Antioxidant Activity of Essential Oils from Selected *Pinus* Species from Bosnia and Herzegovina

**DOI:** 10.3390/antibiotics14070677

**Published:** 2025-07-03

**Authors:** Snježana Mirković, Milica Martinović, Vanja M. Tadić, Ivana Nešić, Aleksandra Stolić Jovanović, Ana Žugić

**Affiliations:** 1 PHI Hospital “Sveti Vračevi”, Srpske vojske 53, 76300 Bijeljina, Bosnia and Herzegovina; snjezanam1983@gmail.com; 2 Department of Pharmacy, Faculty of Medicine, University of Nis, Boulevard Dr. Zorana Djindjića 81, 18000 Nis, Serbia; milica.martinovic@medfak.ni.ac.rs; 3 Institute of Medicinal Plants Research “Dr. Josif Pančić”, Tadeuša Košćuška 1, 11000 Belgrade, Serbia; ivana.nesic@medfak.ni.ac.rs (I.N.); azugic@mocbilja.rs (A.Ž.); 4 “Filly Farm” Pharmacy, Miloša Velikog bb, 11320 Velika Plana, Serbia; stolic_aleksandra@yahoo.com

**Keywords:** *Pinus*, antimicrobial activity, antioxidant activity, essential oil, pines

## Abstract

Essential oils are lipophilic secondary metabolites produced in various parts of aromatic plants and stored in specialized secretory structures. They play a vital role in plant defense, offering protection against microorganisms and herbivores. These oils are known for a wide range of biological activities, including antibacterial, anti-inflammatory, antitumor, analgesic, antioxidant, and immunomodulatory effects. Given the increasing interest in natural alternatives to synthetic drugs, this review explored the therapeutic relevance of *Pinus*-derived essential oils as promising candidates in modern phytotherapy. Species of the genus *Pinus* have been widely investigated for their phytochemical composition and biological potential, with a focus on their medicinal and pharmaceutical applications. This review aimed to assess the biological properties of *Pinus* species commonly used in traditional medicine. In this paper, thorough insight into the chemical composition, as well as into the antimicrobial and antioxidant activities of essential oils obtained from the different parts of *Pinus* species, was given. Although recognized for their antimicrobial activity against a wide range of bacterial strains, including both Gram-positive and Gram-negative bacteria, the practical application of *Pinus* essential oils is often limited by their physicochemical instability and volatility. Therefore, this review highlighted the advances in formulation strategies, particularly encapsulation techniques, as the possible direction of future research concerning essential oils.

## 1. Introduction

Although modern pharmacy primarily relies on the development of synthetic and semi-synthetic drugs, a growing trend of returning to natural resources in both medicine and nutrition has led to the renewed recognition of medicinal plants and their therapeutic potential [1]. This trend is especially present in the area of increasing antimicrobial resistance to antibiotics. Although synthetic antimicrobial agents remain the first line of treatment for infections, their uncontrolled and irrational use has significantly contributed to the spread of bacterial resistance mechanisms. This has led to increased scientific interest in natural-origin therapeutic substances as an alternative. Medicinal plants are recognized as nearly inexhaustible sources of bioactive compounds with antimicrobial activity [2,3]. In addition, bacteria are less likely to develop resistance to the chemically diverse constituents found in plant-derived extracts [4]. Despite the fact that herbal preparations are generally less potent than synthetic antibiotics, their combination often results in synergistic effects, allowing multi-targeted action [5]. This is in line with a shift that recently occurred in classical medicine from the concept of usage of one drug for one target to polypharmacy approach that uses mixtures of chemical entities, such as plant isolates, to act at several targets simultaneously. Aside from antimicrobial activity, according to the same principle, the usage of medicinal plants is applicable in impairments associated with oxidative stress, while a growing body of research indicated their significant antioxidant potential [6].

**Figure 1:**
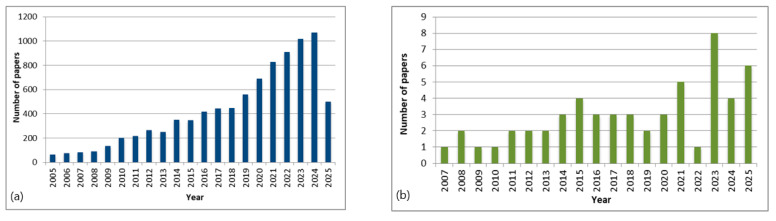
Number of scientific research found on Pubmed in the last 20 years (2005–2025): (**a**) 8951 papers were found when the search was restricted to the terms “essential oil”, “antioxidative”, “antibacterial”, and “antimicrobial” as the keywords; (**b**) 56 papers were found when the search was restricted to the terms “essential oil”, “antioxidative”, “antibacterial”, “antimicrobial”, “pine”, and “pinus” in all fields.

Essential oils are lipophilic secondary metabolites synthesized in different parts of aromatic plants and localized in specialized secretory tissues [7,8], typically obtained through steam distillation [9]. These oils are important for plant survival as they protect against microorganisms, herbivores and insects [10]. Essential oils have been shown to exhibit a range of biological activities, including antibacterial, anti-inflammatory, antitumor, analgesic, antioxidant, and immunomodulatory properties, so they are used to alleviate symptoms of serious diseases such as diabetes, cardiovascular diseases, Alzheimer’s disease, and even cancer [11,12]. In recent years, essential oils have gained significant popularity in the food, pharmaceutical, cosmetic, and agricultural industries [13,14]. The number of studies focusing on the characterization of essential oils is constantly growing, particularly those focusing on antimicrobial and antioxidant activities (Figure 1).

The focus on the production and use of essential oils as important sources of bioactive compounds is reflected in several factors. Essential oils are derived from a wide variety of medicinal and aromatic plants, making them readily available [15]. Conventional methods for isolating essential oils such as steam distillation and its variants (microwave and ultrasound-assisted systems) and cold pressing are simple, cost-effective, and accessible [16]. Innovative methods for isolating essential oils are increasingly being used, which reduce the time and energy spent, and lead to better yield, quality, and bioactive potential of essential oils (e.g., microwave extraction and supercritical fluid extraction) [17]. A significant advantage of essential oils compared to other plant extracts is the minimal use of organic solvents, which are used in the isolation of other plant isolates. Therefore, essential oils as products have a lower risk of residual toxic compounds and increase the safety of the products, especially for pharmaceutical, food, and cosmetic applications. In addition, essential oils possess substantial economic potential, given their strong market demand and diverse industrial applications [18]. Significant amounts of residual biomass generated during essential oil production can be transformed through innovative approaches and advanced technologies into valuable resources such as: flavonoids and phenolic acids, which are used in different industries; hydrosols, as valuable co-products due to their insecticidal, antioxidant, and antibacterial properties; biochar and composite, which are used in soil conditioning, agriculture, and energy production [19].

The main components of essential oils are terpenes and phenylpropanoids—a diverse group of compounds that exhibit strong antimicrobial potential, especially due to their synergistic effects with other antimicrobial agents [10,11,12]. Natural antioxidants from essential oils play a vital role in preventing diseases like diabetes, neurodegenerative, and cardiovascular disorders [20,21,22]. Unlike synthetic antioxidants, which may cause side effects after long-term use, natural alternatives are considered safer [23].

The genus *Pinus* L. (pines) belongs to the family Pinaceae and comprises approximately 110 species, making it the largest genus among gymnosperms. The natural distribution of this genus is confined to the Northern Hemisphere, with species widespread across Asia, Europe, North Africa, and Central America. Most species grow as trees, while a smaller number grow as shrubs, exhibiting high resistance to extreme habitat conditions [24]. Various *Pinus* species have demonstrated a range of biological activities *in vitro*, including antimicrobial, antioxidant, anti-inflammatory, antimutagenic, and anticancer effects. In folk medicine, essential oils isolated from *Pinus* species are commonly used in the treatment of wounds, and disorders of the pulmonary, urinary, or hepatic systems [25]. Hence, there is a notable growth of studies investigating their biological activities in order to justify wide-spread traditional uses.

This review focused on the chemical composition, as well as the antimicrobial and antioxidant effects of essential oils obtained from different parts of *Pinus* species from Bosnia and Herzegovina, specifically *P. mugo* Turra, *P. nigra* J.F., *P. sylvestris* L., *P. heldreichii* Ant., *P. pinea* L., *P. peuce* Griseb., *P. cembra*, *P. halepensis* Miller, and *P. heldreichii* Crist that have numerous ethnomedicinal applications, especially in the treatment of skin and respiratory health impairments [25,26]. Considering the number of investigated species within the *Pinus* genus, and the lack of a concise summary on the selected *Pinus* species from Bosnia and Herzegovina, this review is directed at a survey of the literature’s data considering the stated *Pinus* species. For this purpose, an extensive electronic survey was performed using multiple search platforms such as Scopus, Web of Science, and Medline. The keywords used included: “pinus”, “pine”, “essential oil”, “antimicrobial”, “antibacterial”, and “antioxidant” covering studies published between 2005 and 2025.

## 2. Chemical Composition of Essential Oils from *Pinus* Species

Species of the genus *Pinus* have been the subject of numerous studies focusing on chemical characterization or biological activities of plant isolates from bark and needles for medicinal and pharmaceutical purposes [11,27,28,29,30,31,32]. On the other hand, data on the biological properties and chemical profile of essential oils from green cones are limited [33,34,35]. The wide range of biological activities of pine essential oils is largely attributed to their chemical profile, which includes compounds from various classes of secondary metabolites.

The essential oils derived from different parts of *Pinus* species exhibit complex chemical compositions. Across all species examined, α-pinene is the most consistently dominant compound in both needles and cones. However, essential oils from needles tend to be more complex and diverse, frequently containing limonene, β-pinene, δ-3-carene, bornyl acetate, (*E*)-caryophyllene, myrcene, and various sesquiterpenes like germacrene D and caryophyllene oxide. In contrast, essential oils from cones show higher relative abundances of caryophyllene oxide, aromadendrene, and abietane-type diterpenes such as dehydroabietal and norabietanes, especially in *P. sylvestris* and *P. nigra*. This indicated a greater presence of oxygenated sesquiterpenes and diterpenes in cones, possibly reflecting their more resinous, protective role in securing pine reproductive tissues from insects, microbes, fungi, and other threats [11,35,36,37,38].

The essential oils of *P. sylvestris* and *P. peuce* are characterized by a high content of α-pinene, exceeding 40%, while β-pinene is most abundant in the essential oil of *P. peuce* (13.0%) [39]. The major constituents of *P. peuce* essential oils include α-pinene, β-phellandrene, β-pinene, germacrene D, and α-terpinol, which are linked to their biological activities and, consequently, its potential therapeutic applications in the treatment of inflammation and cancer [40]. The aim of the study by Semerdjieva et al. was to investigate the influence of distillation time on the quantitative and qualitative composition of essential oils obtained from *Pinus* species growing in Bulgaria, with the goal of optimizing essential oils extraction for targeted chemical profiles. The findings indicated that prolonged distillation (2–3 h) is not necessary for the extraction of essential oil from *P. peuce* with a high content of α-pinene, limonene, camphene, and β-pinene [41]. Essential oils derived from different parts of *P. cembra* (the needles, twigs, bark, wood, and cones) were subjected to chemical analysis, resulting in the identification of more than 130 compounds. Notable variations were observed in the quantitative composition of the oils depending on the plant part [42].

An overview of references identifying the chemical profiles of essential oils from different parts of the *Pinus* species studied in this paper is provided in Table 1. Although advanced techniques such as supercritical CO_2_ extraction are increasingly utilized for the extraction of essential oils, primarily due to the non-polar nature of CO_2_, the majority of the reviewed studies employed hydrodistillation as the extraction method. The main reason of hydrodistillation’s dominance over other extraction methods, including supercritical CO_2_ extraction, might be due to practical and economic reasons. Hydrodistillation is conducted using a Clevenger apparatus, which is simple and easy for operation, and relatively low-cost. This method is easy to scale, and water as a renewable and safe solvent is used. On the other hand, supercritical CO_2_ extraction requires specialized high-pressure equipment, higher capital investment, and skilled operation. Due to its high cost, it is not yet suitable for widespread use in the conventional extraction processes and is currently limited to the production of relatively high-value products [43,44].

Furthermore, gas chromatography–mass spectrometry (GC-MS) was predominantly used for the analysis of the chemical composition.

## 3. Antimicrobial Activity of Essential Oils of *Pinus* Species

Essential oils represent potentially useful sources of antimicrobial compounds [76]. When combined with antibiotics, essential oils reduce the harmful effects of synthetic antimicrobial drugs and lower the effective dose of antibiotics in treating infections. Most importantly, these combinations aim to overcome bacterial resistance, where, in addition to synergistic effects, additive ones may also occur [27]. Essential oils from *Pinus* species have been studied for their antimicrobial properties against bacteria and fungi, proving their potential as good sources of antimicrobial agents [29,37,77,78,79,80]. Pine essential oils are known for their antimicrobial activity against a wide range of bacterial strains, including both Gram-positive and Gram-negative bacteria. An overview of references confirming the antimicrobial activity of essential oils and extracts from the different parts of these pine species is presented in Table 2. The results of the microdilution method used to determine antimicrobial activity were expressed as minimum inhibitory concentration (MIC) or minimum bactericidal concentration (MBC), i.e., the lowest concentration of an antimicrobial that inhibits visible microbial growth or kills the microorganisms. The results of disc diffusion method were expressed as the diameter of inhibition zones, indicating antimicrobial activity (Table 2).

The antimicrobial activity of pine essential oils has been attributed to the dominant presence of α-pinene, whose antimicrobial properties have been confirmed in many studies [55,72,83,90]. Essential oils from the needles of four pine species (*P. mugo* subsp. *mugo*, *P. nigra* subsp. *nigra*, *P. sylvestris*, and *P. peuce*) have shown an inhibitory effect on the growth of bacterial strains *K. pneumoniae*, *E. coli*, *S. aureus*, with minimum inhibitory concentration (MIC) values ranging from 1.25 to 20.00 mg/mL [90]. Fatbardhë et al. observed that the essential oil from needles of *P. mugo* and *P. heldreichi* had only week-long activity against *C. albicans*, while the essential oil from *P. sylvestris* showed moderate activity against *C. albicans*. Kurti et al. confirmed that essential oils from *P. mugo* and *P. sylvestris* needles exhibited moderate activity against *C. albicans*, while essential oils from *P. mugo*, *P. sylvestris*, *P. heldreichi*, and *P. nigra* did not inhibit the growth of *E. coli* and *E. faecalis. P. nigrae* essential oil exhibited no antibacterial activity against all tested microorganisms [28].

The essential oil derived from the resins of *P. pinea* cultivated in the Mediterranean region of Turkey exhibited varying degrees of antimicrobial activity against Gram-positive and Gram-negative bacteria, as well as yeast species. These results provide scientific validation for the traditional use and indicate their potential applicability in the development of environmentally friendly pharmaceutical formulations and biological control agents [67] *P. peuce* essential oil exhibited the strongest antimicrobial activity specifically against *L. monocytogenes* and *E. coli* [41].

The essential oils tested by Mitić et al., demonstrated varying levels of efficacy, which can be ranked in the following descending order: *P. sylvestris* > *P. peuce* > *P. nigra* subsp. *nigra* > *P. mugo* subsp. *mugo*. The essential oil of *P. peuce* exhibited antimicrobial activity, particularly against *M. morganii* with MIC/MBC = 2.50/5.00 mg/mL, as well as against *E. coli* and *S. aureus* strains isolated from the throat (MIC = MBC = 5.00 mg/mL). The observed antimicrobial effects of the tested *Pinus* essential oils was attributed, at least in part, to the dominant presence of α-pinene, as previously reported. However, the comparison between *P. sylvestris* (α-pinene 41.9%, β-pinene 3.2%) and *P. peuce* (α-pinene 49.3%, β-pinene 13.0%) indicates that a higher concentration of a major antimicrobial constituent did not necessarily correlate with increased antimicrobial efficacy. This suggested that the overall activity of essential oils might be influenced by the synergistic or antagonistic interactions among their various chemical components [39]. 

The chemical composition, and consequently biological activity, of essential oils are influenced by factors such as environmental conditions (plant growing location, soil, air temperature during collection, climate, collection time), genetics, sampling techniques, essential oil extraction methods, chromatographic processing, etc. As a result, essential oils derived from plants growing in different habitats or countries may display different phytochemical properties and antimicrobial effects [91]. In addition, there are variations between species. For instance, the antimicrobial activity of essential oil from *P. halepensis* was more pronounced than in the case of *P. pinea*, as the latter showed no activity against *B. subtilis*, *M. lutea*, *P. mirabilis* and *C. albicans* [89]. The essential oil from *P. sylvestris* exhibited a stronger bactericidal effect compared to *P. peuce*, which, in turn, was more effective than *P. nigra* ssp. *nigra* and *P. mugo* ssp. *mugo* [39]. In the study where essential oils from cones and needles of *P. mugo*, *P. nigra*, *P. sylvestris*, and *P. halepensis* from Bosnia and Herzegovina were examined, the best antimicrobial activity was observed for oils of *P. sylvestris* cones, *P. halepensis* cones, and *P. halepensis* needles [25].

Differences in the chemical composition of essential oils may account for the variations in their antibacterial activity. The mechanism of antimicrobial action of terpenes, the main components of essential oils, is linked to their lipophilic properties, which facilitate their penetration through the microbial cell wall. This disrupts membrane integrity, causes coagulation of cellular contents, inhibits efflux pumps, interferes with the respiratory chain, causes potassium ion leakage, reduces expression of genes involved in biofilm synthesis, alters protein structures, etc. [5].

Gram-positive bacteria are generally more susceptible to essential oils due to the oils’ hydrophobic nature, which makes it more difficult for them to penetrate the hydrophilic cell wall of Gram-negative bacteria. This was demonstrated in many studies. For instance, *P. halepensis* and *P. pinea* essential oil was more effective against *B. subtilis* and *M. lutea* (Gram-positive) than against *P. mirabilis*, and *E. coli* (Gram-negative) [89]. Politeo et al. showed that *P*. *nigra* ssp. *dalmatica* needles’ essential oil was almost inactive against enterobacteria *E. coli*, *E. cloacae*, and *K. pneumonia* [29]. However, the comparative study of various *Pinus* essential oils revealed that their antimicrobial activity was not based only on interactions with cell wall structures, but involved mechanism of action at multiple levels [39,84]. *P. mugo* needle essential oil, rich in α-phellandrene, showed a strong antimicrobial effect against Gram-negative *S. enterica* ssp. *enterica*, and *E. coli* [41]. Essential oil from aerial parts of *P*. *halepensis* was effective against *L. monocytogenes*, *K. pneumonia*, *E. faecalis*, and *A. baumanii*, but ineffective against *S. aureus*, *B. cereus*, *E. coli*, *S. typhimurium*, and *P. mirabilis* [37]. Mitić et al. reported the inhibitory effect of the EO isolated from the needles of *P. heldreichii* against *S. aureus*, *K. pneumoniae*, and *E. coli* in concentrations from 1.5 to 12 mg/mL. Among the tested strains, the highest sensitivity was observed in the case of *S. aureus* from a nasal swab. Limonene and α-pinene, the major components of the currently studied oil of *P. heldreichii*, can be considered as compounds at least partially responsible for the observed antimicrobial action against *S. aureus* [87].

As antibiotic resistance is one of the greatest threats to mankind, identifying agents capable of overcoming antibiotic resistance and effectively targeting multidrug-resistant bacterial strains has become of utmost importance. Essential oils, as complex mixture of various compounds, can target multiple bacterial cellular systems at the same time, which is why bacteria are less prone to become resistant to essential oils than to antibiotics, as single molecules [92]. Moreover, it was shown that essential oils can inhibit the action of transmembrane efflux pumps, which represent the main mechanisms of bacterial resistance, as these pumps expel antibiotics from the intracellular space [93]. *P*. *nigra* ssp. *dalmatica* needles’ essential oil showed significant activity against *P. aeruginosa*, well-known multidrug resistant bacteria, which is highly resistant to most antibiotics. The same activity was observed against *C. indologenes* and *A. hydrophila* [29], as well as against *P. aeruginosa* [25].

Biofilms are complex, structured communities of microorganisms enclosed within a self-produced extracellular matrix and attached to either living (biotic) or non-living (abiotic) surfaces. Biofilm formation enhances antibiotic tolerance through various mechanisms, including the retention of antibiotics within the extracellular matrix, metabolic adaptation, moderate expression of efflux pumps, and the induction of a quiescent state [81]. Antimicrobial activity against biofilms is especially important, since most antibiotics are effective only against actively growing microorganisms. The situation is different within biofilms, where due to the reduced nutrient availability, bacteria are less metabolically active. It was demonstrated that *P. mugo* needles’ essential oil could inhibit the formation of biofilm of highly resistant *S. enteroica* [82]. Essential oils from *P. sylvestris* inhibited biofilm formation in *N. gonorrhoeae* at short incubation time in dose-dependent manner. The mechanism proposed for this activity was insertion of α-pinene in lipopolysaccharide layer and consequent membrane disruption [81].

A literature survey confirmed the potential of essential oils isolated from *Pinus* species as prospective substances in the treatment of skin, respiratory, urinary, and gastrointestinal infections, based on the sensitivity of previously tested bacterial/fungal species recognized as causative agents of the stated impairments (Table 2). Different parts of the *Pinus* species have ethnotherapeutic applications in the treatment of skin conditions such as eczema, acne, alopecia, psoriasis, and fungal infections, as well as in wound healing. Topical preparations made from various *Pinus* species are traditionally known to be effective in treating a range of skin disorders, owing to their anti-inflammatory, antiseptic, and antioxidant properties. Chen et al. reported that α-pinene (one of the main constituents of different *Pinus* sp. essential oil) was active against *S. aureus*, *S. epidermidis*, and *P. acnes*, all of which are involved in the pathogenesis of skin infections [94]. Mirković et al. also reported the high antimicrobial activity of essential oils from *P. nigra*, *P. sylvestris*, and *P. halepensis* against *S. aureus* [25].

The studies have shown the potential of *Pinus* essential oils in the treatment of respiratory infections. Essential oil obtained from *P. mugo* needles showed significant activity against Gram-positive bacteria that can cause respiratory infections [82]. Essential oil from *P. sylvestris* needles showed excellent antibacterial activity *K. pneumoniae*, comparable to streptomycin. Compounds that contributed to this activity were α-terpineol, borneol, fenchol, palmitic acid, caryophyllene, oleic acid, and δ-cadinene [46]. In addition, the essential oils extracted from the needles of four *Pinus* species (*P. mugo* ssp. *mugo*, *P. nigra* ssp. *nigra*, *P. sylvestris*, and *P. peuce*) originating from the central Balkans demonstrated promising inhibitory activity against respiratory pathogenic bacterial strains, like *K.pneumoniae*, *E. coli*, *M. morganii*, and *S. aureus* isolated from human swabs [39]. Essential oils of different *Pinus* species from Bosnia and Herzegovina, rich in α-pinene, (*E*)-caryophyllene, and myrcene displayed strong antibacterial effects with MICs in the range of 100–600 μg/mL against *E. coli*, and showed potential in the treatment of gastroenteritis and urinary tract infections [25]. The results of the study of Mitić et al. suggest that diterpene alcohol thunbergol may serve as a significant antimicrobial agent, either on its own or through synergistic interactions with other compounds [87].

In addition, essential oils are interesting candidates for application in food industry as natural preservatives. This refers especially to those with low inhibitory concentrations. Since essential oils can also contribute to food aroma, they should be effective at low concentrations [85]. Essential oil obtained from *P. mugo* needles was examined in the vapour phase on apple and beetroot model as potential bactericide. This oil showed potential as food preservative against foodborne pathogens *E. aerogenes* and *P. putida* [82]. Essential oil from *P. halepensis* cones was effective against plant bacterial isolates, *Agrobacterium tumefaciens*, *Dickeya solani*, *Pectobacterium atrosepticum*, and *Ralstonia solanacearum.* It was stated that this essential oil was especially effective against potato diseases [88].

Several studies have been conducted in which *Pinus* essential oil was compared to essential oils from other plant species that show antimicrobial potential. For instance, seven essential oils of different plant species were analyzed. Essential oils from *P. sylvestris* and *Citrus* × *limon* (L.) Osbeck, Rutaceae, both rich in limonene, showed good activity against *N. gonorrhoeae*, better than essential oils derived from *Cymbopogon martini* (Roxb.) Wats., Poaceae, *Cinnamomum cassia* (L.) J.Presl, Lauraceae, *Melaleuca alternifolia* (Maiden & Betche) Cheel, Myrtaceae, *Eucalyptus globulus Labill.*, *Myrtaceae* and *Origanum vulgare* L., Lamiaceae. However, this was not the case with the *S. suis* strain, against which *O. vulgare* essential oil was the most effective [81]. Essential oil derived from *P. sylvestris* needles showed lower MIC against *S. aureus* than 60 other tested essential oils among which were the oils from *Achillea millefolium* L., Asteraceae, *Salvia officinalis* L., Lamiaceae, *Zingiber officinale* Roscoe, Zingiberaceae, *Carum carvi* L., Apiaceae, *Citrus* × *paradisi* Macfad., Rutaceae and *Helichrysum italicum* (Roth) G. Don fil., Asteraceae [85].

A novel approach in controlling resistant pathogens involves the combined use of drugs [95]. To explore potential strategies for overcoming bacterial resistance, the investigation can be expanded to examine the synergistic effects of various combinations of essential oils from different chemotypes.

It has been established that essential oils act as synergistic enhancers of the antimicrobial activity of antibiotics. Their combination with standard drugs resulted in a synergistic effect that surpassed the individual therapeutic potential of each, leading to improved antimicrobial efficacy [96]. Mechanisms underlying the pharmacological synergism between essential oils and antibiotics included multi-target effects, where compounds simultaneously acted on various bacterial cell sites (enzymes, substrates, metabolites and proteins, receptors, ion channels, transport proteins, ribosomes, DNA/RNA, and physicochemical mechanisms); pharmacokinetic or physiochemical effects, such as improved solubility by adding another compound; increased permeability of the bacterial membrane and more efficient penetration of antibiotics into the cell; the inhibition of enzyme activity (e.g., beta lactamases, coagulases, lipases, amino acid decarboxylases); inhibition of antibiotic resistance mechanisms in bacteria (for example, inhibition of efflux pumps implicated in exporting antimicrobials outside the bacterial cells, inhibition of ATP-ase activity and increased permeability and disruption of the bacterial membrane); inhibition of metabolic pathways in the membrane that lead to changes in the composition of the cell wall, leading to inhibition of respiration, leakage of cell ions, and reduction of proton motive force etc. [97,98,99]. Our recent study revealed synergistic interaction between gentamicin and the essential oils from cones of selected *Pinus* species (*P. nigra*, *P. sylvestris* and *P. halepensis*) against *S. aureus* and *K. pneumoniae*. The results indicated that this combination could enable the significant reduction of gentamicin concentration by as much as 26.6-fold in some cases, while maintaining the same level of bacterial growth inhibition [25]. Additionally, the combination of itraconazole and *P. sylvestris* essential oil demonstrated strong synergistic activity against *Cryptococcus neoformans* [100].

Antimicrobial activity of the investigated essential oils was assessed by microdilution method represented by MIC/MBC values in majority of studies (Table 2), followed by the diffusion method., represented by inhibition zones. The results presented in this section pointed out the wide range of MIC values of the tested essential oils for the same microbial strain. Taking into account that antimicrobial activity was assessed in *in vitro* studies, further *in vivo* investigations are needed for definitive confirmation of their antimicrobial potential that would validate ethnopharmacological application of the investigated pine essential oils.

## 4. Antioxidant Activity

Plants are natural reservoirs of polyphenolic units with proven antioxidant activity that they exert through various mechanisms: they act as free radical “scavengers”, i.e., electron or hydrogen donors, they complex metal ions, they remove reactive oxygen species, convert hydroperoxides into non-radical species, deactivate singlet oxygen, and affect the expression of enzymes (catalase, superoxide dismutase, glutathione reductase, glutathione peroxidase), which are crucial for neutralizing oxidative stress [101,102].

Antioxidant potential can be increased by synergistic interactions or decreased by antagonistic effects between different antioxidant compounds present in a mixture, a mixture of different plant extracts or plant essential oils, synthetic antioxidants and natural products. Examples of synergistic and antagonistic antioxidant actions are as follows: the interaction between phenolic antioxidants produces synergistic effects with the combination of rosmarinic acid and quercetin, or rosmarinic acid and caffeic acid, while antagonistic effects are obtained with α-tocopherol/caffeic acid; the combined mixture of *Mentha spicata* L., Lamiaceae, and honey produced synergistic antioxidant activity; a synergistic antioxidant effect between methanol rosemary extract, and butylated hydroxytoluene, has been proven, etc. [103].

Up-to-date studies have demonstrated that phenolic compounds, as constituents of *Pinus* essential oils, play a key role in scavenging free radicals, thereby mitigating the harmful effects associated with oxidative stress. Notably, the number of these investigations is scarce in comparison to available data regarding antimicrobial potential of these essential oils, which could be expected due to their lipophilic nature [56,104]. Considering the well-documented antioxidant properties of *Pinus* species in many studies, their isolates are believed to be potentially effective in preventing pathological conditions such as atherosclerosis, diabetes, cancer, Alzheimer’s disease, and rheumatoid arthritis. These disorders are often associated with excessive production of free radicals, which cause oxidative damage to biomolecules such as lipids, proteins, and DNA [105].

The studies that investigated the antioxidant potential of selected *Pinus* species essential oils using the following methods: DPPH (2,2-diphenyl-1-picrylhydrazyl) radical scavenging assay, TBA (Thiobarbituric Acid) assay, POCL (Peroxyoxalate Chemiluminescence), β-carotene/linoleic acid bleaching assay are presented in Table 3.

Essential oils contain a wide range of chemical compounds, some of which are non-polar (e.g., terpenes, hydrocarbons) [110]. The DPPH method is not the most suitable method for assessing lipophilic compounds such as essential oils for several reasons. These assays typically use organic solvents such as methanol or ethanol, which can affect the reactivity of antioxidants and may not mimic the behavior of antioxidants in a lipophilic environment such as essential oil [111]. The DPPH test primarily measures antioxidant activity via a single electron transfer mechanism, which may not encompass the full spectrum of antioxidant mechanisms concerning essential oils. Therefore, relying solely on DPPH results may provide an incomplete picture of their antioxidant potential [112,113]. Carotene/linoleic acid method and TBA are useful for studies of lipophilic antioxidants. However, the beta-carotene bleaching test was less widely used than DPPH due to low reproducibility and problems with quantification [114,115].

In relation to various types of *in vitro* methods used to determine the antioxidant activity of certain *Pinus* species, *in vivo* methods have been performed less frequently. Bouzenna et al. conducted an *in vivo* study in rats, which evaluated the effects of *P. halapensis* essential oil on aspirin-induced liver and kidney damage. Based on the results of lipid peroxidation levels (TBARS), activity of superoxide dismutase (SOD), glutathione peroxidase and catalase (CAT), it was concluded that the application of *P. halapensis* essential oil inhibited aspirin-induced liver and kidney damage, confirming the antioxidant potential of this essential oil [116].

The antioxidant potential of essential oils derived from the needles of four Pinaceae species (*P. cembra* L., *P. mugo*, *Picea abies* L., and *Abies alba* M.) was evaluated using DPPH radical scavenging assay. All tested essential oils exhibited concentration-dependent antioxidant activity. Among them, *P. mugo* essential oil demonstrated the highest radical scavenging capacity in both assay systems, outperforming other *Pinaceae* essential oils [84]. In contrast, Kurti et al. reported that the essential oil extracted from the needles of *P. mugo* exhibited relatively low to moderate DPPH radical scavenging activity. *P. sylvestris* essential oil and its fractions were also tested displaying a weak to moderate antioxidant potential. In the same study, the needle essential oils and corresponding fractions of *P. nigra*, *P. peuce*, and *P. heldreichii* were also assessed, revealing generally weak antioxidant activity [28]. Koutsaviti et al. investigated the antioxidant activity of essential oils, as well as organic and hydroethanolic extracts, obtained from fresh needles of 54 *Pinus* species using POCL assay. Among all tested essential oils, those derived from *P. canariensis* and *P. attenuata* exhibited the highest antioxidant activity. Notably, *P. nigra* var. *caramanica* essential oil also demonstrated significant activity. The observed antioxidant potential may be attributed to synergistic interactions among various constituents, particularly terpene derivatives such as germacrene D, β-caryophyllene, and γ-terpinene, which have been known for their radical scavenging properties [106]. The pronounced antioxidant potential of *P. halepensis* essential oil was further substantiated by the findings of Postu et al., who reported significant radical scavenging activity in both DPPH and ABTS/TEAC/Trolox equivalent antioxidant capacity assays. Additionally, *P. halepensis* essential oil demonstrated neuroprotective effects by mitigating amyloid beta (1–42)-induced memory impairment and oxidative stress in the hippocampus of rats, suggesting its potential therapeutic relevance in the context of Alzheimer’s disease [117].

In contrast to the performed studies regarding the antioxidant potential of essential oils from needles of the presented *Pinus* species, there are no available data on this activity from essential oils isolated from cones.

## 5. Formulation Strategies

In recent years, there has been considerable scientific interest in essential oils due to their biological and pharmacological properties, which can be exploited across several fields, including pharmaceuticals, food, and agriculture. In addition to their antibacterial [25,29,55], antifungal [118], and antioxidant activities [17,92,93], essential oils from different genus of the *Pinaceae* family also exhibit insect larvicidal [55], acaricidal [119], herbicidal [120], molluscicidal [121], antiplatelet [122], anti-inflammatory [40], and anticancer effects [123]. However, their widespread application and commercialization remain limited by unfavorable physicochemical properties, such as high volatility, thermal degradation, poor water solubility, and stability issues that may result in a reduction or loss of effectiveness. The development of appropriate formulation strategies offers the opportunity to overcome these limitations. One of the proposed approaches is the encapsulation process based on preparation of aqueous nano-dispersions. Nanocarriers can be broadly classified into two main categories: polymeric nanoparticulate formulations and lipid-based carriers, including liposomes, solid lipid nanoparticles, nanostructured lipid carriers, and both nano- and microemulsions. Among all the suitable platforms, micro- and nanoemulsions have been the most extensively studied, due to the simplicity of formulation, ease of handling, and cost-effectiveness in production [124,125].

An innovative topical formulation (o/w nanoemulsion) containing the antifungal agent, voriconazole and the essential oil of *P. sylvestris* was proposed as a good candidate to treat onychomycosis. The optimized nanoemulsion demonstrated significantly enhanced *in vitro* permeation compared to both voriconazole and the essential oil of *P. sylvestris* when applied individually, indicating a synergistic interaction between the components. Furthermore, *in vitro* antifungal assays confirmed the efficacy of this novel formulation against a clinical strain of *Microsporum canis* [126].

One of the principal constituents of pine essential oil, α-pinene, exhibits a broad range of biological activities. To improve its physicochemical properties, α-pinene was encapsulated in conventional liposomes and in drug-in-cyclodextrin-in-liposomes systems using hydrogenated (Phospholipon 90H) or non-hydrogenated (Lipoid S100) phospholipids. Both carrier types facilitated the sustained release of α-pinene and maintained the DPPH scavenging activity of α-pinene [127]. Khoa Huynh et al. conducted a study in which they developed a novel self-microemulsifying drug delivery system based on pine essential oil has been developed for encapsulating cyclosporin A for the production of soft capsules for oral use. This system improves the solubility and bioavailability of the immunosuppressant cyclosporin A [128].

Pine isolates show great potential for application as natural additives and preservatives in food formulations. The structural changes of chitosan-shellac-based bio-emulsions caused by the incorporation of pine needle essential oil were investigated. The effect of improving the physical, functional and antibacterial properties of the coatings on egg preservation was proven [129]. Novel antimicrobial peptides isolated from *P. densiflora* Sieb. et Zucc. exhibited strong antimicrobial activity against foodborne bacteria [130]. Pine oil-based cleaning products are used in the household to clean and disinfect most surfaces and clothing. Terpenes from pine oil, which have antimicrobial effects, significantly contribute to the ability to deodorize and disinfect areas and objects [131].

The lack of data on *Pinus* species selected to be presented in this review regarding the applications in the food industry and as natural cleaning products highlights a significant necessity for this field of research. On the other hand, the pine needles and cones as waste collected in the forests might represent interesting source of active substances as a basis for formulations that might expand the shelf life of food, or to be used as disinfectant.

## 6. Toxicology Risk Assessment of Essential Oils

In order to expand the application of the investigated *Pinus* species essential oils, a toxicological risk assessment should be conducted, enabling shedding light on the complex interactions between chemicals and their potentially hazardous effects. As essential oils represent a complex mixture of numerous compounds, mainly terpenoids and phenylpropanoids, the toxicological effects might be due to their lipophilicity, which allows them to readily cross the cell wall and cytoplasmic membrane [132]. In higher doses, essential oils might change membrane fluidity, causing their abnormal permeability, which leads to the leakage of radicals, cytochrome C, calcium ions, and proteins. The consequence is the production of secondary reactive oxidizable species in association with primary reactive species, resulting in oxidative stress and bioenergetic failure. The destruction of the cellular and organelle membranes might lead to cell death by apoptosis and necrosis [133,134,135]. The available literature data mainly referred to aliphatic and aromatic hydrocarbons investigated to assess their toxicological potential [136]. The literature survey on several main constituents of the presented essential oils and based on the current existing data revealed that α-pinene does not present a concern for genotoxicity, when the basis was a reference dose of 1.18 mg/kg/day, a predicted skin absorption value of 40%, and a no-expected sensitization induction level for skin sensitization of 7000 μg/cm^2^ [137]. The same conclusion concerns caryophyllene and caryophyllene oxide [138,139]. Based on the existing data, β-caryophyllene and its oxide are not considered skin sensitizers.

Although pure compounds, known as the constituents of essential oils, were evaluated for their genotoxicity, repeated dose toxicity, reproductive toxicity, local respiratory toxicity, phototoxicity/photoallergenicity, skin sensitization, and environmental safety, the data regarding essential oils as a complex mixture are scarce. Recently, Sartori Tamburlin et al. proposed a tiered approach based on the toxicological evaluation of maximized concentrations of the constituent in several investigated essential oils, while the genotoxic potential of each constituent was assessed using the literature data or Quantitative Structure-Activity Relationship (QSAR) analysis [140]. However, to our knowledge, *Pinus* species, particularly those investigated in this study, have not been previously examined with respect to the toxicological properties of their essential oils. This constitutes a potential direction for future investigations.

## 7. Conclusions

The genus *Pinus* represents a valuable source of bioactive compounds with significant therapeutic potential. Essential oils derived from the various parts of *Pinus* sp. exhibited diverse biological activities. Their notable antimicrobial and antioxidant effects are primarily attributed to the presence of monoterpenes, sesquiterpenes, and phenolic compounds such as α-pinene and β-caryophyllene. The antimicrobial efficacy of these essential oils against a broad spectrum of pathogenic microorganisms, including antibiotic-resistant strains and biofilm-forming bacteria, highlighted their relevance as natural alternatives or supplement to conventional antibiotics. Additionally, their antioxidant capacity revealed their potential role in alleviating diseases associated with oxidative stress.

Despite their promising bioactivities, the practical application of *Pinus* essential oils is often limited by their physicochemical instability and volatility. Recent advances in formulation strategies, particularly encapsulation techniques, offered viable solutions to enhance their stability, bioavailability, and therapeutic efficacy. Future research should focus on standardizing extraction methods, conducting detailed toxicological assessments, and evaluating the clinical effectiveness of these formulations.

## Figures and Tables

**Table 1:** Chemical profile of essential oils from different parts of selected *Pinus* species.

Species Needles/Cones	Ref.	Essential Oil Isolation Method	Method of Quantification	Dominant Compounds
*Pinus sylvestris* L. green needles	[45]	industrial distillation	GC-FID, GC-MS	α-pinene (11.2–40.7%), limonene (0.4–9.4%), β-phellandrene (0.3–9.4%), γ-cadinene (1.7–11.0%), germacrene-D-4-ol (0.5–18.7%), β-pinene (1.1–23.7%)
	[11]	hydro- distillation	GC-FID, GC-MS	α-pinene (7–16%), δ-3-carene (5.2–14.3%), bornyl acetate (1.1–3.9%), β-caryophyllene (2.6–5.8%), δ-cadinene (2.3–5.9%), α-cadinol (4.2–8.5%), epi-α-cadinol, epi-α-muurolol, α-muurolol (4.0–9.1%)
	[30]	hydro- distillation	GC-MS	α-pinene (36.21–51.09%), camphene (4.78–10.73%), β-pinene (5.54–17.09%)
	[46]	hydro- distillation	GC-MS	α-terpineol (27.17%), borneol (6.72%), fenchol (3.52%), caryophyllene (1.3%), δ-cadinene (0.23%), palmitic acid (1.32%)
	[47]	hydro- distillation	GC-MS	3-cyclohexan-1-methanol, α,α,4-trimethyl (21.17%); 3-cyclohexen-1-ol, 1-methyl-4-(1-methylethyl) (21.82%); cyclohexanol, 1-methyl-4-(1-methylethenyl) (14.07%); borneol (6.72%); 1,4-methanoazulene, decahydro-4,8,8-trimethyl-9-methylene-[1.α.3a.β,4.α,8a.β] (5.45%); trans-13-octadecenoic acid (4.71%)
	[48]	hydro- distillation	GC-MS	α-pinene (6.1–26.1%), δ-3-carene (4.9–22.9%), bornyl acetate (0.2–5.6%), (*E*)-caryophyllene (2.9–7.9%), δ-cadinene (2.7–8.2%), α-cadinol (3.9–9.8%), epi-α-cadinol, epi-α- and α-muurolol (4.0–9.1%)
*Pinus sylvestris* L. green cones	[35]	hydro- distillation	GC-MS	18-norabieta-8,11,13-triene (15.78%), α-pinene (14.78%), caryophyllene oxide (12.58%), dehydroabietal (7.12%), abieta-8,11,13-triene (5.2%), 19-norabieta-8,11,13-triene (4.75%), norabieta-4(18), 8,11,13-tetraene (4.59%), β-caryophyllene (2.87%)
	[49]	hydro- distillation	GC-FID, GC-MS	aromadendrene (20.2%), α-pinene (18.5%), α-longipinene (10.5%), α-terpineol (5.5%), caryophyllene oxide (3.6%), limonene (3.3%), and pinocarveol (3.0%)
*Pinus mugo* L. needles	[50]	hydro- distillation	GC-FID, GC-MS	α-pinene (12.11–35.10%), α-phellandrene (8.3–27.0%), β-phellandrene (2.9–10.9%), β-caryophyllene (4.2–10.3%)
	[33]	hydro- distillation	GC-FID, GC-MS, ^1^H-NMR	α-pinene (18.6%), 3-carene (11.3%), bornyl acetate (8.3%), camphene (3%), β-pinene (3.1%), myrcene (4.3%), β-phellandrene (3.6%), germacrene (4.6%), limonene (3.8%), terpinolene (4.6%), α-terpinyl acetate (2.8%), (*E*)-β-caryophyllene (2.8%), α-cadinol (3.2%)
	[51]	hydro- distillation	GC-FID, GC-MS	α-pinene (16.9–24.5%), δ-3-carene (15.4–27.8%), E-caryophyllene (4.4–8.9%), germacrene D (4.0–8.3%), γ-terpinolene (2.2–4.3%),β-pinene (1.5–5.4%), limonene+β-phellandrene (1.9–5.9%), bornyl acetate (2.6–8.1%), δ-cadinene (2.1–3.6%)
	[52]	hydro- distillation	GC-MS	α-pinene (33.3%), γ-muurolene (3.5%), α-fenchene (6.4%), γ-cadinene (9.0%), caryophyllene oxide (6.1%), spathulenol (3.5%), α-cadinol (4.4%), T-muurolol (4.6%), α-terpinyl acetate (3.6%)
	[41]	hydro- distillation	GC-MS-FID	α-phellandrene (16.23–23.76%), β-phellandrene (12.13–28.79%), α-pinene (12.68–17.84%)
	[53]	hydro- distillation	GC-FID, GC-MS	car-3-ene (20.70 ± 9.41%), β-phellandrene (16.44 ± 5.48%), α-pinene (14.99 ± 4.25%), myrcene (7.82 ± 8.02%), caryophyllene (5.30 ± 1.40%)
*Pinus mugo* L. green cones	[33]	hydro- distillation	GC-FID, GC-MS, ^1^H-NMP	(*E*)-β-caryophyllene (24%), 3-carene (19.2%), myrcene (16.5%), α-pinene (8.9%), abieta-6(8),14-dien-18-al (4.8%), β-phellandrene (4.5%), α-humulene (3.8%), limonene (3.0%)
	[51]	hydro- distillation	GC-FID, GC-MS	E-caryophyllene (10.4–27%), δ-3-carene (10.5–31.5%), linalool (tr-3.6%), α-pinene (3.1–5.7%), aromadendrene (tr-3.3%), limonene+β-phellandrene (2–9.3%)
*Pinus nigra* J.F., needles	[29]	hydro- distillation	GC-MS	α-pinene (24.3%), β-pinene (16%), germacrene D (14.6%), β-caryophyllene (9.6%)
	[54]	steam distillation	GC-MS	α-pinene (52.19%), germacrene D (14.27%), caryophyllene (5.67%)
	[55]	maceration with organic solvents	GC-FID, GC-MS	α-pinene (45.4%), germacrene D (28.03%), (*E*)-caryophyllene (7.69%), β-pinene (5.47%), germacrene D-4-ol (1.02%)
	[56]	hydro- distillation	GC-FID, GC-MS	caryophyllene oxide (0.99–90.85%), camphene (0.15–38.07%), β-caryophyllene (0.7–42.82%), α-amorphene (0.52–26.04%), germacrene (0.16–27.13%)
*Pinus nigra* J.F., green cones	[35]	hydro- distillation	GC-MS	α-pinene (45.36%), caryophyllene oxide (8.05%), β-caryophyllene (6.73%), dehydroabietal (3.33%), 18-norabieta-8,11,13-triene (3.42%)
*Pinus halapenis* Miller, needles	[57]	maceration with organic solvents	GC-MS	cembrene (33.03%), β-caryophyllene (11.89%), α-pinene (5.30%), myrcene (4.87%), phenylethyl isovalerate (3.99%), methyl-8,13(15)-abietadien-18-oate (2.57%), sabinene (2.41%), terpinolene (2.47%), neoabietol (2.10%), α-humulene (2.03%), methyl levopimarate (1.88%), methyl dehydroabietate (1.81%), neoabietal (1.43%)
	[58]	hydro- distillation	GC-MS	β-caryophyllene (22.20%), cembrene (13.24%), cyclofenchene (8.05%)
	[58]	microwave-assisted extraction	GC-MS	β-caryophyllene (31.50%), α-caryophyllene (7.80%), phenyl isovalerate (6.29%)
	[59]	hydro- distillation	GC-MS	caryophyllene (40.78%), α-pinene (10.30%), dehydro-1,1,7-trimethyl-4-methylene-[1aR-(1aα,4aα,7α,7aβ,7bα)] 1H-cyclopropa[e]azulene (9.53%), 3-carene (7.58%), thunbergol (4.03%), butylphosphonic acid di(2-phenylethyl) ester (3.48%), 3-methylene-bicyclo[3.2.1]oct-6-en-8-ol (4.07%)
	[60]	hydro- distillation	GC-MS	β-caryophyllene (28.04%), myrcene (23.81%), α-pinene (12.02%)
	[61]	unknown	unknown	caryophyllene (48.77%), phenyl isovalerate (22.22%), β-myrcene (15.55%), α-pinene (14.52%)
	[62]	unknown	unknown	β-caryophyllene, myrcene, p-cymene, α-pinene, sabinene, methyl chavicol, methyl iso-eugenol
	[54]	steam distillation	GC-MS	β-caryophyllene (19.05%), α-pinene (13.40%), myrcene (6.62%), cembrene (7.62%), butenoic acid, 3-methyl, 2-phenylethyl ester (6.57%), δ-3-carene (6.87%), limonene (5.03%), terpinolene (3.07%), α-humulene (3.36%)
	[38]	hydro- distillation	GC-MS	(Z)-β-caryophyllene (40.31%), α-humulene (7.92%), aromadendrene (7.1%), myrcene (3.07%), α-pinene (1.23%), sabinene (1.23%)
	[63]	hydro- distillation	GC-FID, GC-MS	longifolene (33.9%), β-pinene (10.70%), α-pinene (9.9%), β-myrcene (9.52%), (Z)-muurrola-4-(14).5-diene (7.40%), α-terpinolene (5.36%), α-humulene (5.20%),
	[64]	hydro- distillation	GC-MS	myrcene (17.5–21.6%), (Z)-β-caryophyllene (17.3–21.2%), p-cymene (7.9–11.9%), caryophyllene oxide (5.4–12.6%), α-pinene (8.5–12.9%)
	[65]	hydro- distillation	GC-MS	caryophyllene (32.97%), α-pinene (13.72%), β-pinene (11.02%), α-humulene (7.68%), cembrene (6.30%), trans-β-ocimene (5.31%), 1R-α-pinene (5%), β-phenylethyl isovalerate (4.43%),
*Pinus halapenis* Miller, green cones	[59]	hydro- distillation	GC-MS	caryophyllene (38.26%), 3-carene (31.78%), 4-methylene-1-(1-methylethyl)-bicyclo[3.1.0]hexane, (11.74%), 1-methyl-4-(1-methylethyl)-1,4-cyclohexadiene (3.01%)
	[66]	hydro- distillation	GC-MS, ^13^C-NMR	α-pinene (47.5%), myrcene (11.0%), (*E*)-β-caryophyllene (8.3%), caryophyllene oxide (5.9%)
	[63]	hydro- distillation	GC-FID, GC-MS	α-pinene (51.70%), α-phellandrene (11.88%), β-myrcene (10.33%), longifolene (15.05%)
	[35]	hydro- distillation	GC-MS	α-pinene (47.09%), β-myrcene (6.25%), β-caryophyllene (11.22%), caryophyllene oxide (7.47%), β-pinene (2.75%), α-humulene (2.65%)
*Pinus peuce* Griseb. fresh needles	[39]	hydro- distillation	GC-MS	α-pinene (49.3%), β-pinene (13%), germacrene D (6.5%), bornyl acetate (7.7%)
	[41]	hydro- distillation	GC-MS-FID	*α*-pinene (34.26–43.75%), limonene (19.2–22.2%), *β*-pinene (9.24–11.48%) (from 0 to 15 min distillation timeframe)
	[40]	hydro- distillation	GC-FID, GC-MS	*α*-pinene (36.8%), *β*-phellandrene (6.1%), *β*-pinene (13%), germacrene D (10%), *α*-terpinol (9.3%), camphene (8%), bornyl acetate (4.2%)
*Pinus peuce* Griseb. twigs	[40]	hydro- distillation	GC-FID, GC-MS	α-pinene (16%), *β*-phellandrene (35.8%), *β*-pinene (21.5%), germacrene D (4.7%); *α*-terpinol (2.4%), camphene (2%), bornyl acetate (3.3%)
*Pinus pinea* L. resin	[67]	hydro- distillation	GC-FID, GC-MS	α-pinene (21.39%), camphene (1.30%), β-pinene (9.68%), D-limonene (5.80%), *β*-phellandrene (1.57%), 1, 4-methenoazulene (8.63%), caryophyllene (9.12%), trans-verbenol (1.76%), α-caryophyllene (2.33%), α-phellandren-8-ol (1.25%), caryophyllene oxide (3.26%)
*Pinus cembra* L. twigs with needles	[42]	hydro- distillation	GC-FID, GC-MS, ^1^H-NMR spectroscopy	*α*-pinene (36.3%), limonene (22.7%), *β*-phellandrene (12.0%), *β*-pinene (4.2%), myrcene (1.0%), camphene (1.0%), *δ*-cadinene (3.8%), methyl lambertianate (0.3%)
*Pinus cembra* L. needles	[42]	hydro- distillation	GC-FID, GC-MS, ^1^H-NMR spectroscopy	*α*-pinene (48.4%), limonene (7.5%), *δ*-cadinene (6.2%), sesquiterpene hydrocarbons (24%)
*Pinus cembra* L. twigs without needles	[42]	hydro- distillation	GC-FID, GC-MS, ^1^H-NMR spectroscopy	limonene (33.6%), *α*-pinene (17.5%), *β*-phellandrene (17.1%), *β*-pinene (7.6%), sesquiterpene hydrocarbons (13%)
*Pinus cembra* L. bark	[42]	hydro- distillation	GC-FID, GC-MS, ^1^H-NMR spectroscopy	limonene (36.2%), *β*-phellandrene (18.8%), *α*-pinene (17.9%)
*Pinus cembra* L. wood	[42]	hydro- distillation	GC-FID, GC-MS, ^1^H-NMR spectroscopy	*α*-pinene (35.2%), *β*-pinene (10.4%), thunbergol (8.4%)
*Pinus cembra* L. unripe cones	[42]	hydro- distillation	GC-FID, GC-MS, ^1^H-NMR spectroscopy	*α*-pinene (35.4%), *β*-pinene (21.6%), limonene (21.1%), oxygenated monoterpenes (1%), oxygenated diterpenes (8%)
*Pinus cembra* L. ripe cones	[42]	hydro- distillation	GC-FID, GC-MS, ^1^H-NMR spectroscopy	*α*-pinene (39.0%), *β*-pinene (18.9%), limonene (3.5%), oxygenated monoterpenes (8%), oxygenated diterpenes (17%)
*Pinus heldreichii* Crist. needles	[68]	maceration with organic solvents	GC-FID, GC-MS	limonene, (19.7%) germacrene D, (25.65%), β-caryophyllene (11.69%), α-pinene (10.14%), Δ3-carene (5.99%)
	[69]	hydro- distillation	GC-FID, GC-MS	limonene, (19.7%) germacrene D, (25.65%), β-caryophyllene (11.69%), α-pinene (10.14%), Δ3-carene (5.99%)
	[70]	maceration with organic solvents	GC-FID, GC-MS	germacrene D (28.%), limonene (27.1%), α-pinene (16.2%), β-caryophyllen (6.9%), β-.pinene (5.2%), β-myrcene (2.3%), pimaric acid (2.0%), α-humulene (1.2%),camphene (0.85%), thunbergo (0.78%), germacreneD-4-ol (0.64%), isopimarol (0.58%), kauran-18-oicacid (0.57%), (*E*)-hex-2-enal (0.52%)
	[71]	hydro- distillation	GC-MS	Limonene (52.8%), germacrene D (15.8%), *α*-pinene (10.2%), *trans*-caryophyllene (7.7%), *β*-pinene (3.0%)
	[72]	maceration with organic solvents	GC-FID, GC-MS	germacrene D (25.1%), α-pinene (19.3%), limonene (14.1%) β-caryophyllene (7.2%), β-pinene (7.0%)
	[73]	hydro- distillation	GC-FID, GC-MS, ^1^H-NMR, IR	limonene (20.26–25.15%), germacrene D (42.6445.42%), β-caryophyllene (10.58–13.32%)
*Pinus heldreichii* Crist. green cones	[74]	hydro- distillation	GC-FID, GC-MS	limonene (75.90–77.75), α-pinene (10.39–12.78), β-myrcene (2.49–2.89), β-cubebene (2.51–2.62), trans-caryophyllene (2.18–2.38)
	[75]	hydro- distillation	GC-FID, GC-MS	Limonene (39.7–81.1%), α-pinen (6.97–21.01%), β-myrcene (1.29–1.75%), β-caryophyllene (0.91–5.83%), germacrene D (0.05–6.99%)

**Table 2:** Overview of the literature’s data on antibacterial activity of essential oils from selected *Pinus* species.

Species	Isolate/ Plant Part	Essential Oil Isolation Method	Test Method	Microorganisms	Results	Ref.
*P. sylvestris*	essential oil from needles	hydro- distillation	microdilution method	*Klebsiella pneumoniae* (nasal and throat swabs), *Escherichia coli* (nasal and throat swabs and sputum), *Morganella morganii* (nasal swab), *Staphylococcus aureus* (nasal and throat swabs and sputum)	MIC = 1.5–10 mg/mL MBC = 2.5–40 mg/mL	[39]
	essential oil from needles	hydro- distillation	microdilution method	*E. coli*, *Candida albicans*, *Enterococcus faecalis*	moderate activity against *C. albicans*; no antibacterial activity against *E. coli* and *E. faecalis*	[28]
	essential oil from needles	hydro- distillation	microdilution method	*Neisseria gonorrhoeae*, *Streptococcus suis*	3 (1–0.06) mg/mL 2 (4–0.12) mg/mL	[81]
	essential oil from needles	hydro- distillation	microdilution method	*S. aureus*, *E. faecalis*, *Kocuria rhizophila*, *E. coli*, *K. pneumonia*, *Salmonella typhimurium*, *C. albicans*	MIC = 0.1–1.0 mg/mL	[25]
	essential oil from cones	hydro- distillation	microdilution method	*S. aureus*, *E. faecalis*, *K. rhizophila*, *E. coli*, *K. pneumonia*, *S. typhimurium*, *C. albicans*, *Pseudomonas aeruginosa*	MIC = 0.1–1.0 mg/mL	[25]
	essential oil from needles	hydro- distillation	disc diffusion method microdilution method	*Bacillus cereus* *S. aureus*, *B. stearothermophilus* *B. subtilis* *E. faecalis* *Micrococcus luteus* *E. coli* *K. pneumoniae* *P. fluorescens*	Inhibition zones: 8–24 mm MIC = 0.025–25.00 mg/mL	[46]
*P. mugo*	essential oil from needles	hydro- distillation	microdilution method	*E. coli*, *C. albicans*, *E. faecalis*	moderate activity against *C. albicans*; no antibacterial activity against *E. coli* and *E. faecalis*	[28]
	essential oil from needles	steam distillation	disc diffusion method microdilution method	Gram-positive bacteria: *Listeria monocytogenes*, *M. luteus*, *S. aureus* Gram-negative bacteria: *Enterobacter aerogenes*, *E. coli*, *P. putida;* Fungi: *C. albicans*, *C. glabrata*, *C. krusei*, *C. tropicalis*	Inhibition zones: 6.67–13.33 mm MIC50 = 2.52–7.41 mg/mL MIC90 = 2.72–7.7 mg/mL	[82]
	essential oil from needles	hydro- distillation	microdilution method	*S. pneumoniae*, *S. pyogenes S. agalactiae*	MIC: 15.26 µL/mL, 7.5 µL/mL, 31.25 µL/mL	[83]
	essential oil from needles	hydro- distillation	microdilution method	*K. pneumoniae* (nasal and throat swabs), *E. coli* (nasal and throat swabs and sputum), *M. morganii* (nasal swab), *S. aureus* (nasal and throat swabs and sputum)	MIC = 2.5–20 mg/mL MBC = 10–40 mg/mL	[39]
	essential oil from needles	steam distillation	microdilution method	Gram-negative bacteria: *P. fluorescens*, *E. coli*, *Acinetobacter bohemicus* Gram-positive bacteria: *Krichia coli*, *P. fluorescens*	MIC = 26.56–52.16 mg/mL	[84]
	essential oil from needles	hydro- distillation	microdilution method	*S. aureus*, *E. faecalis*, *K. rhizophila*, *B. subtilis*, *E. coli*, *K. pneumonia*, *S. typhimurium*, *C. albicans*	MIC = 0.5–1.0 mg/mL	[25]
	essential oil from cones	hydro- distillation	microdilution method	*E. faecalis*, *K. rhizophila*, *E. coli*, *K. pneumonia*, *S. typhimurium*, *C. albicans*	MIC = 0.4–1.0 mg/mL	[25]
	essential oil from needles	industrial distillation	microdilution method	*S. aureus E. coli*	MIC = 0.0–1600 µg/mL	[85]
*P nigra*	essential oil from needles	hydro- distillation	microdilution method	*E. coli*, *C. albicans*, *E. faecalis*	no antibacterial activity against any strain	[28]
	essential oil from needles	hydro- distillation	disc diffusion method microdilution method	Gram-positive bacteria: *B. cereus*, *E. faecalis*, *M. luteus*, *S. aureus;* Fungi: *C. albicans*, *A. niger* Gram-negative bacteria: *Aeromonas hydrophila*, *Chryseobacterium indologenes*, *Enterobacter cloacae*, *E*. *coli*, *K*. *pneumonia*, *P*. *aeruginosa*	Gram-positive: inhibition zones from 0.0 to 37.0 ± 1.5 mm; Gram-negative: inhibition zones from 0.0 to 14.5 ± 0.5 mm; MIC for Gram-positive = 0.03–0.50% (*v*/*v*); MIC for Gram-negative = 0.12–3.2% (*v*/*v*)	[29]
	essential oil from needles	hydro- distillation	microdilution method	*K. pneumoniae* (nasal and throat swabs), *E. coli* (nasal and throat swabs and sputum), *M. morganii* (nasal swab), *S. aureus* (nasal and throat swabs and sputum)	MIC = 2.5–10 mg/mL MBC = 10–40 mg/mL	[39]
	essential oil from needles	industrial distillation	microdilution method	*S. aureus*, *E. coli*	MIC = 0.0–400 µg/mL	[85]
	essential oil from needles	hydro- distillation	microdilution method	*E. faecalis*, *C. albicans*, *K. rhizophila*, *E. coli*, *K. pneumonia*, *S. typhimurium*,	MIC = 0.4–1.0 mg/mL	[25]
	essential oil from cones	hydro- distillation	microdilution method	*E. faecalis*, *C. albicans* *K. rhizophila*, *E. coli*, *K. pneumonia*, *S. typhimurium*,	MIC = 0.1–1.0 mg/mL	[25]
*P. halеpensis*	essential oil from needles	hydro- distillation	paper disc diffusion method	Gram-positive bacteria: *S. aureus*, *B. cereus*, *E. faecalis*, *L. monocytogenes* Gram-negative bacteria: *P*. *aeruginosa*, *E*. *coli*, *S*. *typhimurium*, *A*. *baumanii*, *Citrobacter freundii*, *Proteus mirabilis*, *K*. *pneumoniae*	Inhibition zone 8–10 mm	[37]
	essential oil from needles	unknown	disc diffusion method	*S. aureus*, *P. aeruginosa*, *E. coli*, *B. cereus*	Inhibition zones: 0–4 mm	[86]
	essential oil from needles	hydro- distillation	microdilution method	*E. faecalis*, *C. albicans* *K. rhizophila*, *E. coli*, *B. subtilis*, *K. pneumoniae*, *S. typhimurium*, *P. aeruginosa*	MIC = 0.15–0.8 mg/mL	[25]
	essential oil from needles	hydro- distillation	microdilution method	*K. pneumonia*, *E. coli*, *M. morganii*, *S. aureus*	MIC = 0.19–4.0 mg/mL MBC = 0.5–4.0 mg/mL	[87]
	essential oil from cones	hydro- distillation	microdilution method	*E. faecalis*, *C. albicans* *K. rhizophila*, *E. coli*, *B. subtilis*, *K. pneumoniae*, *S. typhimurium*	MIC = 0.1–0.8 mg/mL	[25]
	essential oil from cones	hydro- distillation	disc diffusion method microdilution method	*B. subtilis*, *Sarcina lutea*, *E. coli*, *S. aureus*	Inhibition zones: 0–12.67 ± 0.58 mm (conc. 125–2000 µg/mL) MIC ≤ 125–2000 µg/mL	[88]
	butanol fraction of essential oil	hydro- distillation	disc diffusion method microdilution method	*B. subtilis*, *S. lutea*, *E. coli*, *S. aureus*	Inhibition zones: 0–15.33 ± 0.58 mm (conc. 125–2000 µg/mL) MIC ≤ 125–>2000 µg/mL	
	essential oil from needles	hydro- distillation	disc diffusion method	*B. subtilis*, *M. lutea* *E. coli*, *P. mirabilis* *C. albican*	Inhibition zones: 8 ± 0.32–39 ± 0.83 mm	[89]
*P. pinea*	essential oils of the resin (hydrodistillation, distilled water)	hydro- distillation	disc diffusion method	*M. luteus*, *B.* *subtilis*, *S. aureus* ATCC 29213, *S. aureus* BAA, *B. cereus*, *E. casseliflavus*, *E. faecalis*, *E. hormaechei*, *E. coli*, *C. albicans*	MIC = 34 mg/mL	[67]
*P. peuce*	essential oil from needles (hydrodistillation, diethyl ether)	hydro- distillation	microdilution method	*K. pneumoniae* (nasal and throat swabs), *E. coli* (nasal and throat swabs and sputum), *M. morganii* (nasal swab), *S. aureus* (nasal and throat swabs and sputum)	MIC = 2.5–20 mg/mL MBC = 5–40 mg/mL	[39]
	essential oil from needles with one-two year old twigs (hydrodistillation, water)	hydro- distillation	disc diffusion method	*S. aureus* subsp. *aureus*, *L. monocytogenes*, *B. cereus*, *S. enterica subsp. enterica*, *P. aeruginosa*, *E. coli*, *C. albicans*, *C. glabrata*, *C. tropicalis*	Inhibition zones: 3.17–8.17 mm	[41]
*P. heldreichii*	essential oil from needles	hydro- distillation	disc diffusion method	Gram-positive bacteria: *S. aureus* subsp. *aureus*, *L. monocytogenes*, *B. cereus* Gram-negative bacteria: *S. enterica subsp. enterica*, *P. aeruginosa*, *E. coli* Fungi: *C. albicans*, *C. glabrata*, *C. tropicalis*	3–8 mm	[41]
	fractions of essential oil from needles (n-hexane fraction, n-hexane/diethyl ether (1:1) fraction, diethyl ether fraction	hydro- distillation	disc diffusion method	*E. coli*, *C. albicans*, *C. krusei* (clinically isolated), *E. faecalis*	*Candida albicans* Inhibition zones: d > 35 mm	[28]
			microdilution method	*E. coli*, *C. albicans*, *C. krusei* (clinically isolated), *E. faecalis*	weak activity against *C. albicans;* inactive against *E. coli* and *E. faecalis*	[28]
	essential oil from needles	hydro- distillation	microdilution method	*S. aureus*, *K. pneumoniae*, *E. coli*	MIC for *S. aureus* = 1.50 mg/mL	[87]

**Table 3:** A review of the literature data on the antioxidant activity of essential oils from needles of selected *Pinus* species.

Species	Essential Oil Isolation Method	Test Method	Results	Reference
*P. sylvestris*	hydrodistillation	POCL	4.86 ± 0.48 μg/mL	[106]
	hydrodistillation	DPPH	0.224 ± 0.011 µgTE/mL EO	[28]
	industrial distillation	DPPH	82.09% inhibition of DPPH radicals (conc. 50 µL/mL)	[107]
	hydrodistillation	DPPH	30.37 ± 2.63 µgTE/mL EO	[108]
	hydrodistillation	DPPH	0.262 ± 0.019 µgTE/mL EO	[28]
*P.nigra*	hydrodistillation	DPPH	0.263 ± 0.021 µgTE/mL EO	[28]
	hydrodistillation	DPPH	2.6 ± 0.1–12.1 ± 1.7% (conc. 0.2–1.0 mg/mL)	[104]
	steam distillation	DPPH	3.08 ± 0.65 µg/mL	[84]
	hydrodistillation and solid phase micro-extraction	β-carotene bleaching test	IC_50_ = 1.59 ± 0.01 µg/mL	[109]
*P. halеpensis*	hydrodistillation	POCL	1.78 ± 0.17 μg/mL	[106]
	hydrodistillation	β-carotene bleaching test	IC_50_ = 45.22 µg/mL	[64]
*P. pinea*	hydrodistillation	DPPH	52.10%	[67]
*P. mugo*	steam distillation	TBA	IC_50_ = 2.42 ± 0.2–4.14 ± 0.3 mg/mL	[36]
*P. heldreichii*	hydrodistillation	DPPH	91.3% inhibition of DPPH radicals	[75]
	hydrodistillation	DPPH	0.141 ± 0.011 μgTE/mLEO)	[28]
	hydrodistillation	DPPH	EC_50_ for aglycones: 0.7 g/L EC_50_ for essential oil: undetermined (maximum concentration only 6% of DPPH)	[71]

## Data Availability

Data available in a publicly accessible repository.
